# A Communication Anti-Jamming Scheme Assisted by RIS with Angular Response

**DOI:** 10.3390/e25121638

**Published:** 2023-12-09

**Authors:** Jinpeng Wang, Wenyu Jiang, Kaizhi Huang, Xiaoli Sun

**Affiliations:** 1 School of Cyber Science and Engineering, Zhengzhou University, Zhengzhou 450001, China; wangjp8023@foxmail.com; 2 Institute of Information Technology, PLA Strategic Support Force Information Engineering University, Zhengzhou 450001, China; jwy513@hotmail.com (W.J.); lgdxsunxiaoli@sina.com (X.S.)

**Keywords:** channel capacity, reconfigurable intelligent surface, anti-jamming, angular response

## Abstract

By optimizing the reconfigurable intelligent surface (RIS) reflection coefficients, the channel capacity of legitimate users can be increased, thereby enhancing the anti-jamming performance of communication systems. However, existing studies on RIS-assisted anti-jamming assume that there is no coupling between the RIS reflection coefficients and the incident angle of electromagnetic (EM) waves, which is quite unreasonable. Therefore, we consider the effect of the incident angle of EM waves on the reflection coefficients of the RIS and propose a communication anti-jamming scheme assisted by an RIS with angular response. Specifically, a problem is formulated to optimize the RIS reflection coefficients so that the legitimate signal is amplified, but the jamming signal is attenuated, thus enhancing the legitimate channel capacity. However, the coupling of the EM incident angle and the RIS reflection coefficients causes the problem to be non-convex. To tackle this problem, we equivalently transform the RIS reflection coefficients optimization problem into a quadratically constrained quadratic programming (QCQP) problem using the complex Taylor expansion and the multidimensional complex quadratic transform (MCQT) and solve it utilizing the alternating direction method of the multipliers (ADMM) algorithm. The simulation results reveal that, compared to other schemes supported by RIS without angular response, the proposed scheme is able to achieve a significant improvement in the anti-jamming performance.

## 1. Introduction

Wireless communication has developed rapidly due to its convenience, flexibility, and adaptability to meet the needs of society for mobile communication. However, due to the broadcast nature of electromagnetic (EM) waves and the openness of wireless channels, legitimate communication channels are highly susceptible to jamming, which can lead to a decrease in channel capacity and subsequently affect the communication performance of the system. Specifically, malicious jammers intentionally send jamming signals to increase the channel’s equivocation for legitimate users. The increase in channel equivocation significantly reduces the channel capacity of legitimate channels, affecting normal communication and information transmission and even leading to the paralysis of the communication system. Therefore, research on highly reliable anti-jamming solutions is essential.

In recent years, various techniques have been developed to enhance the anti-jamming performance, e.g., the direct sequence spread spectrum (DSSS), frequency hopping (FH), power control, and beamforming. The DSSS expands the information signal across a wide frequency band to enhance channel capacity, thereby reducing the communication’s requirement for signal interference plus noise ratio (SINR) [1,2]. The authors in [1] proposed a fast acquisition method for DSSS systems. This method enhances the receiver’s sensitivity by prolonging the coherent integration time. In [2], a novel random positioning DSSS (RP-DSSS) scheme is proposed to enhance the anti-jamming performance. Moreover, FH avoids jamming attacks by rapidly switching communication frequencies [3,4]. In [3], Zhang et al. proposed a novel reinforcement learning (RL)-assisted FH method to balance the synchronization overhead and the anti-interference performance. In order to improve the generalization in various jamming scenarios, Shi et al. proposed an efficient FH scheme based on exponential modulation [4]. However, DSSS and FH are ineffective against full-band jamming attacks and are spectrally inefficient. The power control improves the channel capacity by increasing the SINR at the receiver, which results in improved jamming immunity. Compared to FH, power control is an effective method for combating full-band jamming attacks, and it allows more of the spectrum to be used. In anti-jamming relay communication networks, Feng et al. investigated the discrete power control problem using the Stackelberg game [5]. In [6], the authors proposed a deep deterministic policy gradient (DDPG) to control the power. However, due to the limited transmission power, power control is difficult when dealing with high-power jamming attacks. Moreover, by aligning the beam null with the jamming signal, beamforming can suppress and eliminate the jamming signals. In vehicular networks, the authors propose a cooperative anti-jamming beamforming scheme to solve the control channel jamming problems [7]. The authors in [8] proposed a robust beamforming algorithm and combined it with the compressed sensing theory to reduce the algorithm’s complexity. However, beamforming consumes the resources of the sender and receiver in exchange for its anti-jamming capability, which requires a large number of transceiver antennas and complex signal processing.

A reconfigurable intelligent surface (RIS) consists of a large number of passive low-cost reflective elements, and each element has the ability to independently adjust the amplitude and phase of the signal. Consequently, RIS can be programmed to superimpose and/or cancel signals in a desired manner at the receiver, which provides low energy and low costs yet is a highly spectrally efficient method of anti-jamming. In [9], the RIS was first applied in the field of communication jamming mitigation, and the authors utilized reinforcement learning to jointly optimize the RIS phase to maximize the received SINR. The studies in [10,11] focus on unmanned aerial vehicles (UAVs)-assisted terrestrial communication scenarios and optimized RIS the deployment position and RIS reflection coefficients to enhance the anti-jamming capability. In RIS-assisted UAV communication scenarios, the authors jointly optimized UAV flight trajectories and RIS reflection coefficients to enhance the anti-jamming capability [12,13]. In addition, Sun et al. designed RIS reflection coefficients under imperfect jamming channel state information (CSI) and also achieved significant anti-jamming outcomes [14].

These aforementioned studies heavily rely on the assumption that there is no coupling between the RIS reflection coefficient and the incident angle of the EM wave. However, the experimental results in [15] reveal that the adjustable phase range of the RIS shrinks sharply when the incident angle continues to grow. This trend means that the RIS produces different responses to signals from different incident angles (i.e., angular response). In reality, neglecting the effect produced by angular response leads to a mismatch between the theoretical study and the physical realization of RIS-assisted communication, which greatly reduces the actual anti-jamming performance of the communication system. Additionally, although the existing RIS-based communication system studies consider the impact of angular response, the relevant contribution is limited, not considering the RIS-assisted communication anti-jamming scenario. Motivated by the above reason, we propose a communication anti-jamming scheme assisted by an RIS with an angular response. The specific tasks are as follows:

- Based on the actual model of RIS, the general system model for RIS-assisted communication anti-jamming is established. In this model, the RIS reflection coefficients of incident signals are modeled, and the effect between the reflection coefficient and the incident angle is considered.
- To improve the anti-jamming performance of RIS with angular response, it is necessary to optimize the base station (BS) precoding and RIS reflection coefficients. On the one hand, the BS should design transmission precoding to reasonably allocate the power and phase on different antennas. On the other hand, the RIS reflection coefficients should ensure that the signals of both the direct link and the reflection link are superimposed in the desired manner. Therefore, a joint optimization problem is formulated to acquire the BS precoding and RIS reflection coefficients.
- By taking the SINR as the quality of anti-jamming performance metrics, a problem is formulated to maximize the SINR. Specifically, the BS precoding is acquired by applying the Cauchy–Schwarz inequality. To tackle the non-convexity problem resulting from the coupling of the incident angle and RIS reflection coefficients, the RIS reflection coefficient optimization problem is able to be equivalently transformed into a quadratically constrained quadratic programming (QCQP) problem using the complex Taylor expansion and multidimensional complex quadratic transform (MCQT), which can be solved by alternating the direction method of multipliers (ADMM) algorithm.
- The simulation results reveal that, compared to other schemes supported by the RIS without angular response, the proposed scheme is able to achieve a significant improvement in anti-jamming performance.

## 2. System Model

**Figure 1:**
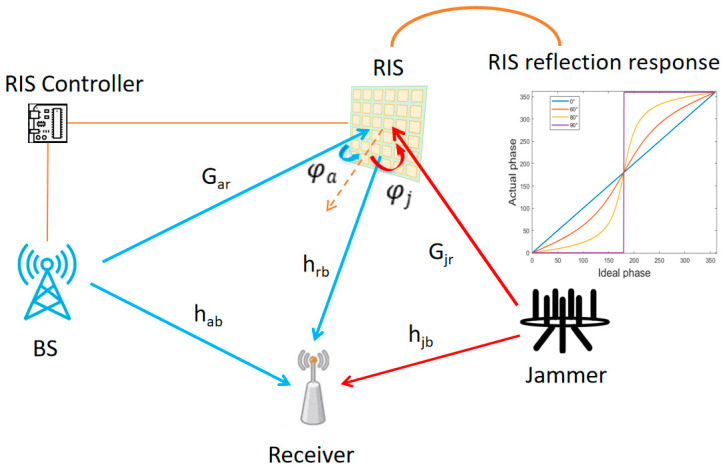
System model.

In this paper, we consider the RIS-assisted multiple-input single-input (MISO) anti-jamming communication scenario, as shown in Figure 1. Among these, the BS equipped with N antennas communicate with a single antenna receiver. The RIS consists of M reflective elements and is controlled by the BS via the RIS controller. The signal reaches the receiver through the direct and reflective links, respectively. During the propagation of this signal in the reflective link, it is first incident on the RIS at the angle of $\varphi_{a}$, then modulated by the RIS, and finally reflected to the receiver. Meanwhile, a malicious jammer sends a jamming signal through L antennas to jam the normal reception in the same way. In this process, the RIS controller dynamically modifies each element to accomplish the reverse cancellation of the jamming signal and in-phase combination of the desired signal at the receiver. Due to the coupling between the reflection coefficient and the incident angle, the EM model of the RIS with angular response needs to be established.

### 2.1. EM Model of RIS with Angular Response

RIS is a reflective array composed of a large number of passive elements, and each element is able to passively reflect incident signals with a controllable phase shift. By appropriately adjusting the phase shift of the passive elements, RIS can achieve reflection-type beamforming with high array gains. As a result, the RIS works as a helper between the transmitter and the receiver to enhance communication performance. The RIS element consists of passive components such as capacitors and inductors. In contrast, both massive multiple-input multiple-output (MIMO) and relays need to be equipped with expensive and powerful radio frequency (RF) components. In addition, since RIS only reflects signals and does not need to receive them, the spacing of RIS array elements does not need to follow the spatial sampling theorem. Compared to massive MIMO, the RIS can accommodate more array elements and achieve high beamforming gains.

**Figure 2:**
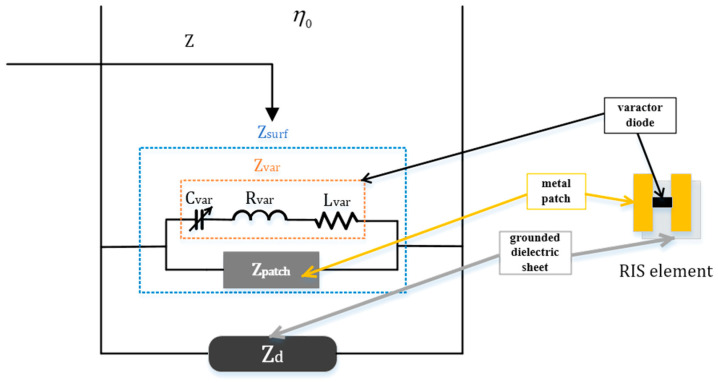
Equivalent circuit model of RIS.

The RIS element consists of two layers, as shown on the right side of Figure 2. The surface layer consists of two symmetric metal patches and a variable capacitor diode. The bottom layer is a grounded dielectric sheet. According to [13,14], the operating mechanism of the RIS element can be elucidated through the application of the equivalent circuit model theory, as shown on the left side of Figure 2. The input impedance of the RIS element Z is obtained using the parallel combination of the surface impedance $Z_{surf}$ and the impedance of the grounded dielectric sheet $Z_{d}$

(1) $$Z=\frac{Z_{surf}Z_{d}}{Z_{surf}+Z_{d}},$$

where $Z_{surf}$ can be represented as the parallel connection of $Z_{\mathrm{var}}$ and $Z_{patch}$ [16].

(2) $$Z_{surf}=\frac{Z_{patch}Z_{\mathrm{var}}}{Z_{patch}+Z_{\mathrm{var}}},$$

where the $Z_{\mathrm{var}}$ and $Z_{patch}$ are the equivalent impedance of the varactor diode and the impedance of the metal patch, respectively.

The $Z_{\mathrm{var}}$ can be represented as a series combination of resistance $R_{\mathrm{var}}$, reactance $L_{\mathrm{var}}$ and variable capacitance $C_{\mathrm{var}}$

(3) $$Z_{\mathrm{var}}=R_{\mathrm{var}}+j\omega L_{\mathrm{var}}+\frac{1}{j\omega C_{\mathrm{var}}},$$

where ω represents the angular frequency.

Once the input impedance of the RIS element Z is determined, the expression for the reflection coefficient Γ can be stated as follows [16]:

(4) $$\Gamma =\frac{Z-\eta_{0}\cos\varphi }{Z+\eta_{0}\cos\varphi },$$

where φ represents the angle between the incident signal and the RIS normal and $\eta_{0}=377\;\Omega$ is the characteristic impedance of free space.

Substituting Equations (1)–(3) into Equation (4), the reflection coefficient can be transformed as

(5) $$\Gamma \left(C_{\mathrm{var}},\varphi \right)=\frac{\left(a-b\cos\varphi \right)C_{\mathrm{var}}+\left(c-d\cos\varphi \right)}{\left(a+b\cos\varphi \right)C_{\mathrm{var}}+\left(c+d\cos\varphi \right)},$$

(6) $$a=Z_{patch}Z_{d}\left(R_{\mathrm{var}}j\omega -\omega^{2}L_{\mathrm{var}}\right),\;$$

(7) $$b=\eta_{0}\left(Z_{\mathrm{patch}}R_{\mathrm{var}}j\omega -Z_{\mathrm{patch}}\omega^{2}L_{\mathrm{var}}+Z_{\mathrm{patch}}Z_{d}j\omega +Z_{d}R_{\mathrm{var}}j\omega -Z_{d}\omega^{2}L_{\mathrm{var}}\right),$$

(8) $$c=Z_{\mathrm{patch}}Z_{d},$$

(9) $$d=\eta_{0}\left(Z_{\mathrm{patch}}+Z_{d}\right).$$

Once the composition and structure of the RIS elements have been determined, the equivalent circuit elements ($Z_{\mathrm{patch}}$, $Z_{d}$ and so on) remain constant. Hence, the reflection coefficient Γ is only related to the equivalent capacitance $C_{\mathrm{var}}$ and the incident angle φ. The RIS can adjust the reflection coefficient of the RIS element by controlling the equivalent capacitance of the varactor diode.

### 2.2. Channel Model

This subsection details the channel model of the communication system. Due to the high path loss in the system, the power of signals reaching the RIS after multiple reflections can be neglected. In addition, to analyze the theoretically improved anti-jamming performance brought by the RIS, we assume that the channel state information (CSI) can be accurately detected and obtained. The channels from BS to RIS, from the jammer to RIS, from BS to receiver, from the jammer to receiver, and from RIS to receiver are denoted as $G_{jr}\in {\mathbb{C}}^{M\times L}$, $G_{ar}\in {\mathbb{C}}^{M\times N}$, $h_{ab}\in {\mathbb{C}}^{N\times 1}$, $h_{jb}\in {\mathbb{C}}^{L\times 1}$, $h_{rb}\in {\mathbb{C}}^{M\times 1}$. The RIS is typically deployed in an optimized position to establish an LOS link between the BS and the RIS, aiming to reduce the impact of RIS multiplicative fading. Additionally, we assume that the link between the jammer and the RIS is also an LOS link, considering the worst-case immunity of the system. Therefore, we model the small-scale fading of the channels $h_{ab}$, $h_{jb}$ and $h_{rb}$ as Rayleigh fading, and the small-scale fading of $G_{ar}$ and $G_{jr}$ as Rician fading with a Rician factor λ=10 used to characterize the LOS communication. Here, $\varphi_{a}$ denotes the angle between the LOS path of the legitimate signal and the RIS normal and $\varphi_{j}$ denotes the angle between the LOS path of the jamming signal and the RIS normal.

### 2.3. Signal Model

When the RIS panel structure is determined, the reflection coefficients for the legitimate and jamming signals are denoted as $\Phi_{a}≜\mathrm{diag}(\Gamma_{a1},\ldots ,\Gamma_{am},\ldots ,\Gamma_{aM})\in {\mathbb{C}}^{M\times M},$ and $\Phi_{j}≜\mathrm{diag}(\Gamma_{j1},\ldots ,\Gamma_{jM})\in {\mathbb{C}}^{M\times M}$, respectively. Among them, $\Gamma_{am}=\Gamma \left(C_{m},\varphi_{a}\right)$, $\Gamma_{jm}=\Gamma \left(C_{m},\varphi_{j}\right)$ and diag(⋅) represents a diagonal matrix obtained through vector diagonalization. In this scenario, the received signal can be represented as follows:

(10) $$y=(h_{ab}^{H}+h_{rb}^{H}\Phi_{a}G_{ar})w_{a}s_{a}+(h_{jb}^{H}+h_{rb}^{H}\Phi_{j}G_{jr})w_{j}s_{j}+n,$$

where $w_{a}\in {\mathbb{C}}^{N\times 1}$ and $s_{a}$ represent the BS precoding vector and data signals, respectively. Similarly, the jamming signal is $w_{j}s_{j}\in {\mathbb{C}}^{L\times 1}$ via the jammer transmitting. In addition, $n\sim CN(0,\sigma^{2})$ represents additive white Gaussian noise, which is a complex Gaussian random variable with 0 mean and variance $\sigma^{2}$. Accordingly, the SINR at the receiver can be represented as follows:

(11) $$\gamma =\frac{{\left|\left(h_{ab}^{H}+h_{rb}^{H}\Phi_{a}G_{ar}\right)w_{a}\right|}^{2}}{{\left|\left(h_{jb}^{H}+h_{rb}^{H}\Phi_{j}G_{jr}\right)w_{j}\right|}^{2}+\sigma^{2}}.$$

## 3. Maximize SINR Scheme

In this paper, we maximize the SINR by jointly optimizing the RIS reflection coefficients and BS precoding, which is subject to the reflection coefficient and the transmission power constraints. Then, the SINR maximization problem can be formulated as follows:

(12) $$\begin{array}{c} P0:\underset{w_{a},\Phi_{s}}{\max}\;f_{0}\left(w_{a},\Phi_{s}\right)=\frac{{\left|\left(h_{ab}^{H}+h_{rb}^{H}\Phi_{a}G_{ar}\right)w_{a}\right|}^{2}}{{\left|\left(h_{jb}^{H}+h_{rb}^{H}\Phi_{j}G_{jr}\right)w_{j}\right|}^{2}+\sigma^{2}}\\ s.t.\;\|w_{a}{\|}^{2}\leq P_{max},\\ \Phi_{s}(m,m)=\Gamma_{sm},\forall m, \end{array}$$

where $P_{max}$ represents the maximum power of the BS, and s∈{a,j}. P0 is a non-convex problem with complex coupling between $w_{a}$ and $\Phi_{s}$ and the constraints of the RIS reflection coefficients. An alternating optimization (AO) algorithm is employed to solve this problem, which decomposes the problem P0 into two sub-problems: (1) optimize BS precoding $w_{a}$ under a fixed RIS reflection coefficient $\Phi_{s}$ and (2) optimize the RIS reflection coefficient $\Phi_{s}$ under fixed BS precoding $w_{a}$. The variables are iteratively optimized alternately until convergence is reached.

### 3.1. Optimize BS Precoding under Fixed RIS Reflection Coefficient

In this section, we investigate the optimization $w_{a}$ under a fixed $\Phi_{s}$. For easier expression, we further define $h_{a}^{H}=h_{ab}^{H}+h_{rb}^{H}\Phi_{a}G_{ar}$. In addition, due to the fact that the term ${\left|\left(h_{jb}^{H}+h_{rb}^{H}\Phi_{j}G_{jr}\right)w_{j}\right|}^{2}$ in problem P0 does not involve $w_{a}$, this term can be treated as a constant. Then, problem P0 can be reformulated as follows:

(13) $$\begin{array}{c} P1:\underset{w_{a}}{\max}\;{\left|h_{a}^{H}w_{a}\right|}^{2}\\ s.t.\;\|w_{a}{\|}^{2}\leq P_{max}. \end{array}$$

The BS precoding vector $w_{a}$ can be given by the following:

(14) $$w_{a}=\sqrt{P_{a}}{\tilde{w}}_{a},$$

where $P_{a}$ is the transmission power, and ${\tilde{w}}_{a}$ is the optimal unit-norm precoding vector. Hence, problem P1 can be reformulated as

(15) $$\begin{array}{c} P1:\underset{w_{a},P_{a}}{\max}\;P_{a}{\left|h_{a}^{H}{\tilde{w}}_{a}\right|}^{2}\\ s.t.\;P_{a}\leq P_{max},\\ {\left\|{\tilde{w}}_{a}\right\|}^{2}=1. \end{array}$$

It is evident that $P_{a}=P_{max}$ is the optimal solution for P1. Then, by utilizing the Cauchy–Schwarz inequality, we could obtain the following:

(16) $${\tilde{w}}_{a}^{opt}=\underset{{\left\|{\tilde{w}}_{a}\right\|}^{2}=1}{\arg\max}\;|h_{a}^{H}{\tilde{w}}_{a}{|}^{2}=\frac{h_{a}}{\left\|h_{a}\right\|}.$$

Substituting Equation (16) and $P_{a}=P_{max}$ into Equation (14), we obtained the optimal transmission precoding vector of the BS

(17) $$w_{a}^{opt}=\sqrt{P_{max}}\frac{h_{a}}{\left\|h_{a}\right\|}.$$

This vector maximizes the SINR and improves system performance under the given transmission power constraint.

### 3.2. Optimize RIS Reflection Coefficient under Fixed BS Precoding

In this section, the optimization of the RIS reflection coefficients $\Phi_{s}$ is investigated under the condition of fixed BS precoding vectors $w_{a}$. For subsequent analysis, we further define $H_{arb}≜diag(h_{rb}^{H})G_{ar}w_{a}$, $h_{ab}≜h_{ab}^{H}w_{a}$, $H_{jrb}≜diag(h_{rb}^{H})G_{jr}w_{j}$, $h_{jb}≜h_{jb}^{H}w_{j}$, $\phi_{a}≜{\left(\Gamma_{a1},\ldots ,\Gamma_{aM}\right)}^{H}$, $\phi_{j}≜{\left(\Gamma_{j1},\ldots ,\Gamma_{jM}\right)}^{H}$. Under the fixed $w_{a}$, the optimization problem can be formulated as

(18) $$\begin{array}{c} P3:\underset{\phi_{s}}{\max}f_{3}\left(\phi_{s}\right)=\frac{{\left|h_{ab}+\phi_{a}^{H}H_{arb}\right|}^{2}}{{\left|h_{jb}+\phi_{j}^{H}H_{jrb}\right|}^{2}+\sigma^{2}},\\ s.t.\;\phi_{s}(m,m)=\Gamma_{sm},\forall m,s\in \{a,j\}. \end{array}$$

P3 essentially represents a high-dimensional fractional programming problem, which can be extended to a matrix form using the MCQT. This transformation effectively resolves the non-convexity issue [17]. Hence, Lemma 1 is obtained as below.

**Lemma** **1.** *With the auxiliary variable* μ *introduced by the MCQT method, P3 can be rewritten as*

(19) P4:maxϕ s f4ϕs,μ=2Re{μH(hab+ϕaHHarb)}−|μ|2hjb+ϕjHHjrb2+σ2s.t. ϕs(m,m)=Γsm,∀m,s∈{a,j}.

**Proof.**  See Appendix A. □

Similarly, the AO algorithm can decompose P4 into two sub-problems and obtain feasible solutions through alternating optimization.

(1) Fix $\phi_{s}$ to find the optimal μ: In this case, $f_{4}\left(\phi_{s},\mu \right)$ is a concave differentiable function that is concave over μ. Therefore, the optimal $\mu^{opt}$ is obtained via $\partial f_{4}\left(\phi_{s},\mu \right)/\partial \mu =0$ and expressed as follows:
  (20) $$\mu^{opt}=\frac{\left(h_{ab}+\phi_{a}^{H}H_{arb}\right)}{{\left|h_{jb}+\phi_{j}^{H}H_{jrb}\right|}^{2}+\sigma^{2}}.$$
(2) Fix μ to find the optimal $\phi_{s}$: ${\left|h_{jb}+\phi_{j}^{H}H_{jrb}\right|}^{2}$, which can be rewritten as
  (21) hjb+ϕjHHjrb2=ϕjHHjrbHϕj+2Re{hjbHϕjHHjrb}+hjb2.

By bringing (21) and (20) into (19) and ignoring the constant term, problem P4 can be rewritten as follows:

(22) P5:maxϕ sf5ϕs,μ=2Re{ϕaHA}−ϕjHΨϕj−2Re{ϕjHΒ}s.t. ϕs(m,m)=Γsm,∀m,s∈{a,j}.

where A, Ψ, and Β are

(23) $$A=\mu^{H}H_{arb},$$

(24) $$\Psi =|\mu {|}^{2}H_{jrb}H_{jrb}^{H},$$

(25) $$B=|\mu {|}^{2}h_{jb}^{H}H_{jrb}.$$

P5 remains a non-convex problem due to the coupling between the incident angle and the RIS reflection coefficient. But, the mapping relationship of the reflection coefficient between different signal incident angles is correlated and can be expressed as follows:

(26) $$\Gamma_{s}=\frac{\left(\cos\varphi_{o}+\cos\varphi_{s})\Gamma_{o}+(\cos\varphi_{o}-\cos\varphi_{s}\right)}{\left(\cos\varphi_{o}-\cos\varphi_{s})\Gamma_{o}+(\cos\varphi_{o}+\cos\varphi_{s}\right)},$$

where $\Gamma_{o}$ is the reflection coefficient when the incident angle of the signal is zero. However, the fractional form eliminates the possibility of solving this via the substitution of variables. To solve this problem, we introduced the complex Taylor expansion to approximate $\Gamma_{s}$, which can be formulated as

(27) $$\Gamma_{s}=Q(\Gamma_{0})=\alpha +\beta \sum_{k=1}^{\infty }{\left(-\alpha \Gamma_{o}\right)}^{k},$$

(28) $$\alpha =(\cos\varphi_{o}-\cos\varphi_{s})/(\cos\varphi_{o}+\cos\varphi_{s}),$$

(29) $$\beta =-4\cos\varphi_{o}\cos\varphi_{s}/({\left(\cos\varphi_{o}\right)}^{2}+{\left(\cos\varphi_{s}\right)}^{2}).$$

Substituting (27) into (22), the optimization problem P5 can be transformed to the following:

(30) P6:maxϕo f6ϕo=2Re{Qa(ϕo)HA}−Qj(ϕo)HΨQj(ϕo)−2Re{Qj(ϕo)HB}s.t. ϕo(m)=1,∀m.

By removing some high-order terms, we can obtain an acceptable approximation. Thus, P6 can be formulated as

(31) P7: maxϕof7ϕo=2Re{ϕoHO}−ϕoHPϕos.t. ϕo(m)=1,∀m.

where O and P are

(32) $$O=-\alpha_{a}\beta_{a}A+\alpha_{j}\beta_{j}B+\alpha_{j}^{2}\beta_{j}\Psi Z,$$

(33) $$P=\alpha_{j}^{2}\beta_{j}^{2}\Psi ,$$

(34) $$Z={\left[1,\cdots ,1\right]}^{T}\in {\mathbb{R}}^{M\times 1}.$$

Undoubtedly, P7 is a QCQP problem, which can be solved using the semidefinite relaxation (SDR) method. However, the SDR method incurs significant computational overhead, with a complexity of approximately $O{\left(M+1\right)}^{6}$, which may hinder the practical application of the anti-jamming design. In order to reduce the computational complexity, we adopted the ADMM method [18] to solve P7.

To utilize the ADMM method, we introduced a slack variable $v\in {\mathbb{C}}^{M\times 1}$ and transform problem P7 as shown below:

(35) P8:maxv f8v=2Re{vHO}−vHPvs.t. v−ϕo=0,ϕo(m)=1,∀m.

Then, the augmented Lagrange function of P7 can be expressed as

(36) L(ϕo,v,λ)=2Re{vHO}−vHPv−ρ2∥v−ϕo∥2+Re{λH(v−ϕo)},s.t. ϕo(m)=1,∀m.

where $\lambda ={\left[\lambda_{1},\cdots ,\lambda_{M}\right]}^{H}$ are the Lagrange variables and *ρ* > 0 is the penalty parameter. The ADMM method ADMM obtains the optimal solution by iteratively updating the dual variables until convergence, which consists of the following three steps:

(37) $$\begin{array}{c} \phi_{o}{}^{t+1}=\underset{\phi_{o}}{\mathrm{argmax}}\;L(\phi_{o},v^{t},\lambda^{t}),\\ v^{t+1}=\underset{v}{\mathrm{argmax}}\;L(\phi_{o}{}^{t+1},v,\lambda^{t}),\\ \lambda^{t+1}=\lambda^{t}-\rho (\phi_{o}{}^{t+1}-v^{t+1}), \end{array}$$

where t is the iteration index. The ADMM method can obtain a closed-form solution in each step of the solution process.

(38) $$\phi_{o}{}^{t+1}=(\rho v^{t}-\lambda^{t}){\left(\left|\rho v^{t}-\lambda^{t}\right|\right)}^{-1},$$

(39) $$v^{t+1}={\left(2P+\rho I\right)}^{-1}\left(2O+\lambda^{t}+\phi_{o}{}^{t+1}\right),$$

(40) $$\lambda^{t+1}=\lambda^{t}-\rho (\phi_{o}{}^{t+1}-v^{t+1}),$$

The specific derivation can be found in Appendix B.

### 3.3. Overall AO-Based Algorithm

Based on Section 2.1 and Section 2.2, this section gives the AO algorithm for joint BS precoding and the RIS reflection coefficient optimization, as shown in Algorithm 1. Among these, the ADMM method computes the inverse of the matrices in the QCQP by (39), which is the main complexity of the AO algorithm. Hence, the algorithm complexity can be approximated as $O\left(I_{m}\times I_{n}\times M^{3}\right)$, where $I_{m}$ is the number of iterations for the alternating optimization of BS precoding and RIS reflection coefficients and $I_{n}$ is the number of iterations for the convergence of the RIS reflection coefficient.

**Algorithm 1:** Optimize the P0 of the proposed AO algorithm**Input:** the CSI of channels $h_{ab},\;G_{ar},\;h_{jb},\;G_{jr},\;h_{rb}$ and the power of the BS and jammer $p_{a},\;p_{j}$.

**Output:**
optimal $w_{a}^{opt}$ and $\Phi_{o}^{opt}$. 
1.

q←1;

2.Initialize $w_{a}^{q}\;\mathrm{and}\;\Phi_{o}^{q}$;3.Initialize $\gamma^{t}$ by (10);4.Initialize err1=1;5.
**while (** err1>0.001 **) performs**
6.

q=q+1

7.Update μ with given $w_{a}^{q}$ by (19);8.t←1;9.Initialize $\lambda^{t}$ and $v^{t}$;10.Calculate $\phi_{o}^{t}$ with given $\Phi_{o}^{q}$;11.Initialize err2=1;12.
**while (** err2>0.001 **) performs**
13.t=t+1;14.Update $\phi_{o}^{t+1}$ with given $\lambda^{t}$ and $v^{t}$ by (38);15.Update $v^{t+1}$ with given $\lambda^{t}$ and $\phi_{o}^{t+1}$ by (39);16.Update $\lambda^{t+1}$ with given $v^{t+1}$ and $\phi_{o}^{t+1}$ by (40);17.

$err2=\left(f_{p6}\left(\phi_{o}^{t+1}\right)-f_{p6}\left(\phi_{o}^{t}\right)\right)/f_{p6}\left(\phi_{o}^{t+1}\right);$

18.
**End;**
19.Calculate $\Phi_{o}^{q+1}$ with given $\phi_{o}^{t+1}$;20.Update $w_{a}^{q+1}$ with given $\phi_{o}^{q+1}$ by (17);21.Update $\gamma^{q+1}$ with given $\phi_{o}^{q+1}$ and $w_{a}^{q+1}$ by (11);22.$err=(\gamma^{q+1}-\gamma^{q})/\gamma^{q+1}$;23.
**End.**



## 4. Simulation Results

In this section, we validate the effectiveness of the proposed scheme through a simulation. We take SINR as the evaluation metric to analyze the impact of the incident angle of the jamming signal, the power of the jammer, the number of IRS elements, and the power of BS. Then, we set the BS at [0, 0] m, and the receiver was located at [50, 0] m. To minimize the performance degradation due to RIS multiplicative fading, we deployed the RIS at [50, 5] m. In addition, considering the equality between the jammer and the BS, we deployed the jammer at [7, −25] m to ensure an equal distance between the jammer and the BS from the receiver. Additionally, the large-scale fading model is considered in this paper, and the distance-dependent path-loss model is given by the following:

(41) $$PL=L_{0}{\left(d/d_{0}\right)}^{-\varepsilon }$$

where d is the distance, $L_{0}=-30\;\mathrm{dB}$ is the path loss at the $d_{0}=1\;m$, and ε is the path-loss exponent [7]. More detailed parameters are shown in Table 1.

We compare the proposed scheme with the following benchmark schemes. **(1) No angular response scheme in** [19]**:** This scheme adopts the method in [19], where the jamming is considered as jamming from other BSs. This scheme jointly optimizes the BS precoding and RIS reflection coefficients to enhance the SINR for legitimate users. In addition, the RIS reflection coefficients are designed based on an ideal model without considering the effect of angular response. **(2) Direct link MRT scheme in** [20]**:** This scheme adopts the method in [20], where the BS precoding is set as $w_{a}=\sqrt{p_{a}}\frac{h_{ab}}{\left\|h_{ab}\right\|}$ after the optimal phase shift is obtained. In addition, the effect of the signal incident angle on the RIS response is considered in the phase shift optimization process. **(3) Minimized jamming scheme in** [21]**:** This scheme employs the method in [21], where the RIS adjusts the reflection coefficient to minimize the strength of the jamming signal at the receiver. In this scenario, the scheme optimizes BS precoding and RIS reflection coefficients to minimize the SINR at the receiver.

**Figure 3:**
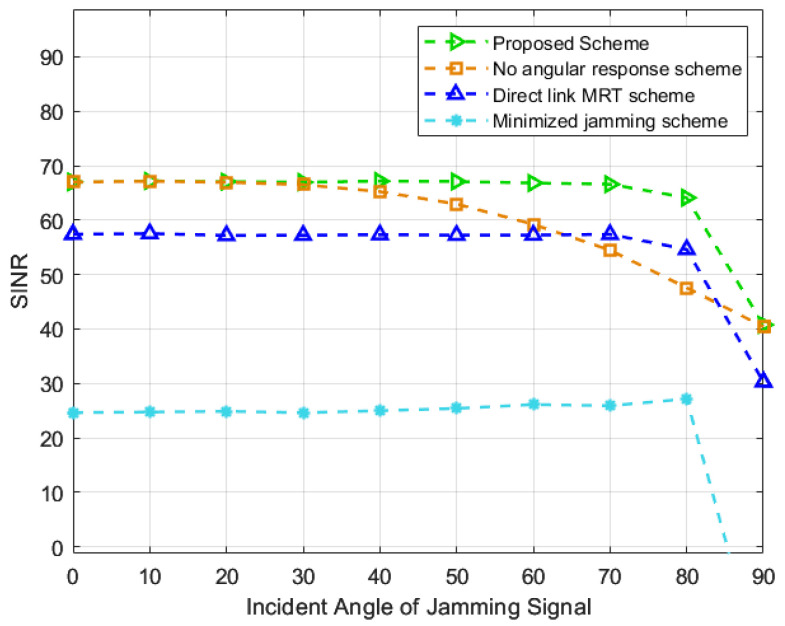
SINR against the incident angle of the jamming signal [19,20,21].

Firstly, to validate the effect of the incident angle of the jamming signal on the SINR, we set the BS at [50, −45] m, which is perpendicular to the RIS reflecting surface. The initial position of the jamming device is located at [50, −45] m, and it moves with the RIS position as the center and a radius of 50 m. As shown in Figure 3, when the incident angle of the jamming signal is zero, both the proposed scheme and the scheme in [19] exhibit the same SINR. As the angle increases, the SINR of the proposed scheme does not decrease significantly, but the SINR of the scheme in [19] drops quickly. The reason for this is that the range of the RIS phase shift decreases as the angle increases. Due to the scheme in [19], the angular response is not considered, and the SINR drops rapidly. On the contrary, the proposed scheme is able to find the optimal phase shift within the RIS phase shift range so that there is no significant decrease in SINR. Additionally, when the angle reaches 90°, there is a sudden drop in SINR for the proposed scheme. The reason for this is that the reflection coefficients of the jamming signal are either −π or π when the angle of incidence is 90°. In other words, the RIS loses its phase-shifting capability, making it impossible to achieve the desired jamming cancellation between the reflected path and the LOS path. As a result, the jamming mitigation performance of the proposed scheme is compromised. Additionally, when the incident angle of the signal is less than 40°, there is almost no performance difference between the scheme in [19] and the proposed scheme. In other words, the RIS response is not sensitive to the incident angle of the signal when it is less than 40°. Therefore, the proposed scheme exhibits better performance gain when the incident angle is greater than 40°.

**Figure 4:**
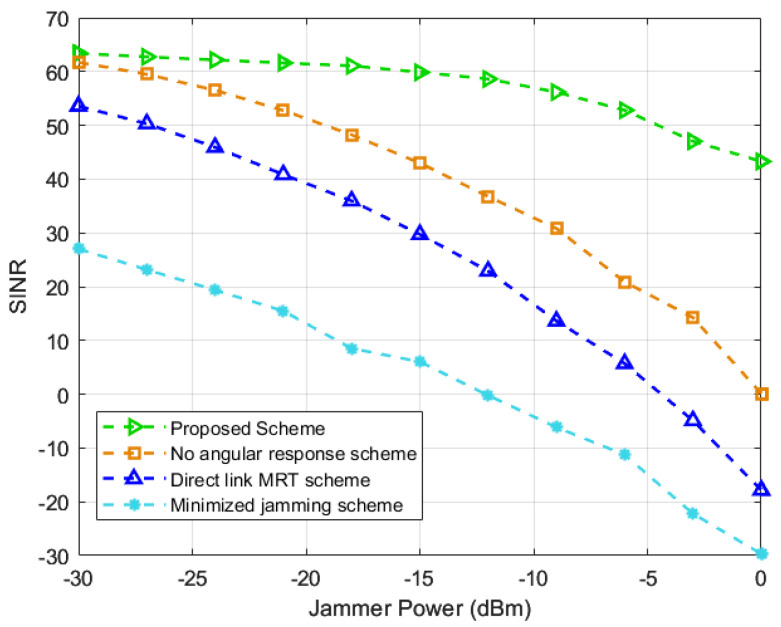
SINR against the power of the jammer [19,20,21].

Moreover, in Figure 4, the influence of the power of the jammer is studied. As shown in the graph, the proposed scheme has the highest SINR. Specifically, the SINR of the proposed scheme is almost the same as that of the scheme in [19], and the minimized jamming scheme is much lower than that of other schemes under low jamming power. The reason for this is that under the low jamming power, the RIS mainly enhances the SINR by boosting the useful signal. The scheme in [19] only utilizes RIS to suppress jamming signals without enhancing the useful signal. Therefore, under lower interference power, the scheme in [19] significantly outperforms other schemes. In addition, with the increase in the jamming power, the SINR of the proposed scheme decreases significantly slower than other schemes. The superiority is verified in the performance of the proposed scheme under high power jamming.

**Figure 5:**
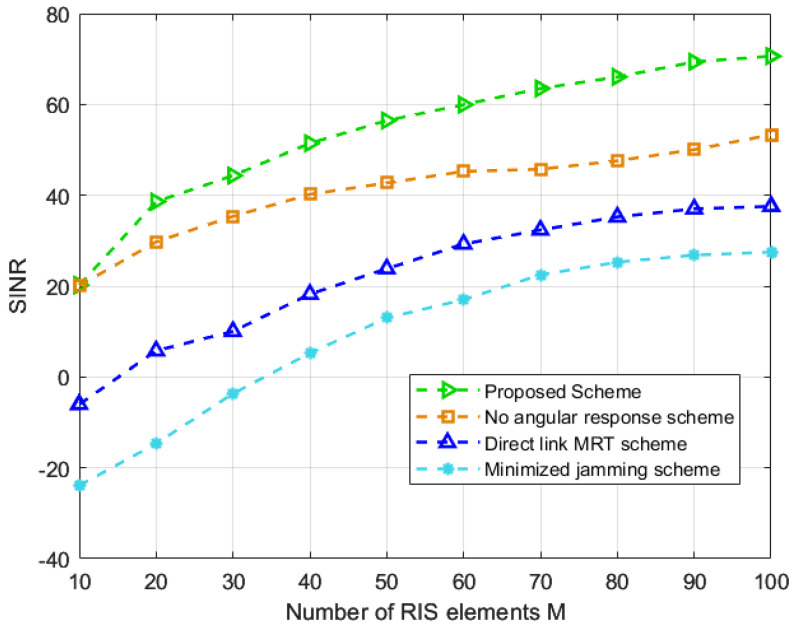
SINR against the number of RIS elements [19,20,21].

Figure 5 shows the relationship between SINR and the number of RIS elements. It can be observed that all schemes show an increasing trend in SINR with an increase in the number of RIS elements, and the proposed scheme has the highest SINR. The reason behind this is that the RIS provides additional spatial degrees of freedom for optimization, and the upper limit of the jamming suppression capability offered by these spatial degrees of freedom increases with the number of RIS elements. However, in all the schemes, the rate of increase in SINR decreases with the increase in the number of RIS elements. Therefore, it is necessary to strike a balance between the cost of RIS and its performance gain in practical applications and make choices based on actual requirements. In addition, the highest SINR of the proposed scheme proves that the proposed scheme can better utilize the gain provided by RIS compared to others.

**Figure 6:**
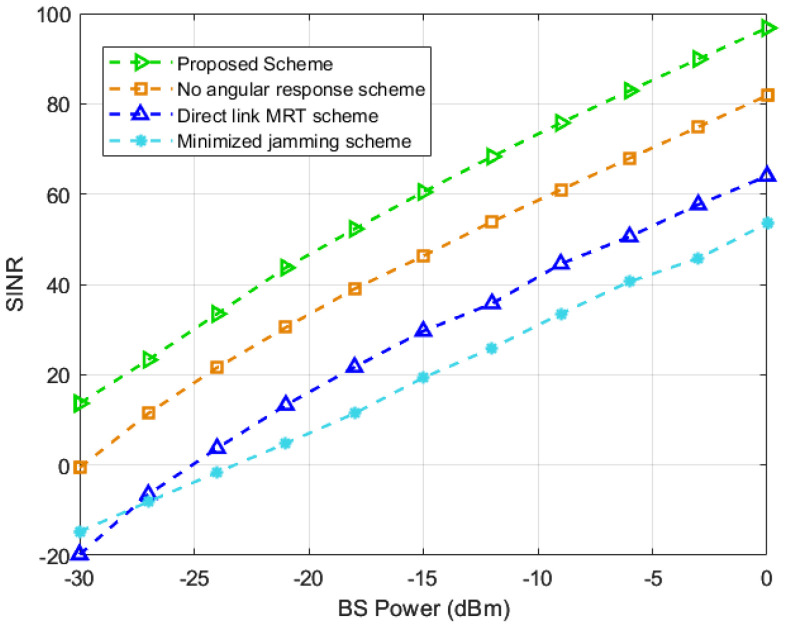
SINR against the power of BS [19,20,21].

Finally, in order to verify the anti-jamming capability of the proposed scheme under different levels of transmitting power, we investigated the effect of the BS transmitting power on SINR. As shown in Figure 6, the growth trend of SINR for all schemes is almost the same when the BS transmitting power increases, and the proposed scheme has the highest SINR. Specifically, the proposed scheme has more than 15dB of gain compared to the scheme in [19] at different levels of BS power and proves the superior anti-jamming performance of the proposed scheme. In addition, the limited performance improvement of the MRT scheme compared to the proposed scheme and scheme in [19] indicates that the BS precoding design is not optimal, and the anti-jamming capability is degraded. Therefore, well-designed BS precoding can effectively improve the anti-jamming performance.

## 5. Conclusions

In this paper, we propose a communication anti-jamming scheme assisted by an RIS with an angular response. Specifically, a problem is formulated for the joint optimization of BS precoding and RIS reflection coefficients so that the jamming signal is attenuated, but the legitimate signal is amplified at the receiver. Then, we apply an AO algorithm to decouple the joint optimization problem into two sub-problems. The BS precoding is solved using the Cauchy-Schwarz inequality, and the RIS reflection coefficient optimization problem is equivalently transformed into a solvable QCQP problem via the MCQT. Simulation results show that the proposed scheme assisted by an RIS with angular response obtains a significant anti-jamming performance improvement, thus demonstrating the practicality of the proposed scheme. Future work should concentrate on the study of joint anti-jamming schemes under imperfect CSI conditions.

## Figures and Tables

**Table 1:** Simulation parameter.

Parameter	Value		
	BS	Receiver	Jammer
Carrier frequency	3.15 GHz		
Number of antennas	4	1	4
RIS configuration	M = 60, $R_{\mathrm{var}}=0.3\mathrm{OHm}$, $L_{\mathrm{var}}=0.7\mathrm{nH}$, $C_{\mathrm{var}}=\left[0.6,\;2.6\right]\mathrm{pF}$		
Path-loss exponent	$\varepsilon_{ab}=3.75$, $\varepsilon_{jb}=3.75$, $\varepsilon_{ar}=2.2$, $\varepsilon_{jb}=2.5$, $\varepsilon_{rb}=2.2$		
Other factors	$\sigma^{2}=-90\;\mathrm{dBm}$, $p_{a}=-15\;\mathrm{dBm}$, $p_{j}=-15\;\mathrm{dBm}$		

## Data Availability

The data are contained within the article.

## Appendix A

**Proof of Lemma** **1.** Let $\Lambda =h_{ab}+\phi_{a}^{H}H_{arb}$ and $\Omega ={\left|h_{jb}+\phi_{j}^{H}H_{jrb}\right|}^{2}+\sigma^{2}$; then, each term in $f_{p4}\left(\phi_{s},\mu \right)$ can be rewritten as $\Lambda^{H}\Omega^{-1}\Lambda -\left(\mu -\Omega^{-1}\Lambda^{H}\right)\Omega \left(\mu -\Omega^{-1}\Lambda \right)$ by completing the square. It can be proved that the optimal solution is $\mu =\Lambda \Omega^{-1}$. Bringing it into P4 proves the equivalence of problem P4 and problem P3. □

## Appendix B

Firstly, the problem of optimizing $\phi_{o}^{t+1}$ based on the given $v^{t}$ and $\lambda^{t}$ can be written as follows:

(A1) P9: maxϕot+1 f9ϕot+1= Re{λH(vt−ϕot+1)}−ρ2∥vt−ϕot+1∥2,s.t. ϕot+1(m)=1,∀m.

To facilitate the handling of the formula, we rewrite the function (A1) as

(A2) P9: maxϕot+1 f9ϕot+1=Reρvt−λHϕot+1−ρ2vt2−ρ2ϕot+12+ReλHvt,s.t. ϕot+1(m)=1,∀m.

Due to the unit modulus of $\phi_{o}^{t+1}(m)$, the term ${\left\|\phi_{o}^{t+1}\right\|}^{2}$ is equal to M. Hence, by removing the constant term, the function (A2) can be rewritten as follows:

(A3) P9: maxϕot+1 f9ϕot+1=Reρvt−λHϕot+1,s.t. ϕot+1(m)=1,∀m.

The optimal solution of $\phi_{o}^{t+1}$ can be obtained via

(A4) $$\phi_{o}^{t+1}=(\rho v^{t}-\lambda^{t}){\left(\left|\rho v^{t}-\lambda^{t}\right|\right)}^{-1},$$

Secondly, based on the fixed $\phi_{o}^{t+1}$ and $\lambda^{t}$, the problem of optimizing $v^{t+1}$ can be expressed as follows:

(A5) P10: maxvt+1 f10vt+1=2Re{(vt+1)HO}−(vt+1)HPvt+1−ρ2∥vt+1−ϕot+1∥2+Re{λH(vt+1−ϕot+1)},

Function $f_{10}\left(v^{t+1}\right)$ is a convex differentiable function of $v^{t+1}$. The optimal $v^{t+1}$ can be obtained by taking the derivative, and the closed-form solution of $v^{t+1}$ can be expressed as follows:

(A6) $$v^{t+1}={\left(2P+\rho I\right)}^{-1}\left(2O+\lambda^{t}+\phi_{o}{}^{t+1}\right),$$

where I is the identity matrix.

Lastly, according to (37), the closed-form solution of $\lambda^{t+1}$ can be expressed as follows:

(A7) $$\lambda^{t+1}=\lambda^{t}-\rho (\phi_{o}^{t+1}-v^{t+1}).$$

As a result, we can obtain the closed-form solutions for each step of ADMM:

(A8) $$\phi_{o}^{t+1}=(\rho v^{t}-\lambda^{t}){\left(\left|\rho v^{t}-\lambda^{t}\right|\right)}^{-1},$$

(A9) $$v^{t+1}={\left(2P+\rho I\right)}^{-1}\left(2O+\lambda^{t}+\phi_{o}^{t+1}\right),$$

(A10) $$\lambda^{t+1}=\lambda^{t}-\rho (\phi_{o}^{t+1}-v^{t+1}).$$
